# Effects of Intravascular Photobiomodulation on Insomnia, Muscle Soreness, and Biochemistry Profiles: An Eight-Year Retrospective Cohort

**DOI:** 10.3390/medicina59061006

**Published:** 2023-05-24

**Authors:** Yen-Po Lin, Ruei-Sian Ding, Chun-Hao Yin, Yao-Shen Chen, Jin-Shuen Chen, Shin-Tsu Chang

**Affiliations:** 1 Department of Medical Education, Kaohsiung Veterans General Hospital, Kaohsiung 813, Taiwan; 2 Department of Medical Education, National Cheng Kung University Hospital, Tainan 704, Taiwan; 3 Department of Physical Medicine and Rehabilitation, Kaohsiung Veterans General Hospital, Kaohsiung 813, Taiwan; 4 Institute of Health Care Management, National Sun Yat-sen University, Kaohsiung 804, Taiwan; 5 Department of Administration, Kaohsiung Veterans General Hospital, Kaohsiung 813, Taiwan; 6 Department of Physical Medicine and Rehabilitation, Tri-Service General Hospital, School of Medicine, National Defense Medical Center, Taipei 114, Taiwan

**Keywords:** intravascular photobiomodulation, pharmacotherapies, blood parameters

## Abstract

Background: Although cognitive-behavioral therapy is the first-line treatment for insomnia, pharmacotherapy is often prescribed to treat insomnia and related symptoms. In addition, muscle relaxants are commonly prescribed to alleviate muscle soreness when the pain is unbearable. However, pharmacotherapy can lead to numerous side effects. The non-drug strategy intravascular laser irradiation of blood (iPBM) has been advocated to improve pain, wound healing, blood circulation, and blood cell function to relieve insomnia and muscle soreness symptoms. Therefore, we assessed whether iPBM improves blood parameters and compared drug use before and after iPBM therapy. Methods: Consecutive patients who received iPBM therapy between January 2013 and August 2021 were reviewed. The associations between laboratory data, pharmacotherapies, and iPBM therapy were retrospectively analyzed. We compared patient characteristics, blood parameters, and drug use within the three months before the first treatment and the three months after the last treatment. We also compared the changes before and after treatment in patients who received ≥10 or 1–9 iPBM treatments. Result: We assessed 183 eligible patients who received iPBM treatment. Of them, 18 patients reported insomnia disturbance, and 128 patients reported pain in any part of their body. After the treatment, HGB and HCT significantly increased after treatment in both the ≥10 and 1–9 iPBM treatment groups (HGB *p* < 0.001 and *p* = 0.046; HCT *p* < 0.001 and *p* = 0.029, respectively). Pharmacotherapy analysis revealed no significant differences in drug use before and after treatment, though drug use tended to decrease after iPBM. Conclusions: iPBM therapy is an efficient, beneficial, and feasible treatment that increases HGB and HCT. While the results of this study do not support the suggestion that iPBM reduces drug use, further larger studies using symptom scales are needed to confirm the changes in insomnia and muscle soreness after iPBM treatment.

## 1. Introduction

According to the World Health Organization, insomnia is highly prevalent and affects one in four adults worldwide [1]. This sleep disorder impairs cognitive and physical functioning and has a significant negative impact on an individual’s work, physical and social performance, as well as their overall quality of life [2,3]. Muscle pain and fatigue are also common problems in general medicine and neurology [4]. Muscle pain and fatigue may coexist and can significantly reduce muscle performance during exercise [5]. These symptoms can often be debilitating and are associated with significant adverse consequences for physical health and wellbeing. Therefore, it is important that effective treatments are provided in clinical practice. The treatment options for the above symptoms include both a variety of pharmacologic therapies and non-medication strategies. Reviews of the available research indicate that pharmacotherapeutic interventions have therapeutic effects in patients with these symptoms, and the risk/benefit profiles of these treatments have been well characterized [6,7,8].

However, medication use and choice is an important concept in medical care because drugs can have many possible adverse effects on multiple organ systems. Non-steroidal anti-inflammatory drugs, which are antipyretic, anti-inflammatory, and analgesic drugs, and the drugs commonly used for insomnia have been reported to cause many side effects [9,10]. In addition, long-term administration of certain drugs, such as opioid analgesics, is associated with high rates of addictive-like behavior and poor recovery outcomes [11,12]. Moreover, long-term administration of hypnotic agents can lead to drug tolerance, dependence, and rebound insomnia [13]. Therefore, non-pharmacologic options may potentially provide longer-lasting benefits and reduce the potential burden of these conditions. In addition, approximately 70% of patients suffering from insomnia in Taiwan are willing to try non-drug therapies [14].

Recently, the term photobiomodulation (PBM) has replaced the previous low-level laser therapy due to it improperly representing electricity and total energy utilization. PBM acts as a form of light therapy by using nonionized light sources to initiate a non-thermal effect in which endogenous chromoplasts result in photo-physicochemical cascades at varying biological levels [15,16]. Intravascular PBM (iPBM) has been advocated since the 1960s; this non-drug therapy was first applied for the treatment of cardiovascular disease and is believed to improve blood circulation and blood cell function [17]. iPBM is considered an innocuous and useful laser therapy technique, particularly when systemic effects are required [18]. iPBM therapy is currently used as an adjunct for pain management in patients with conditions such as diabetic neuropathy, fibromyalgia, and arthritis [19,20,21]. In addition, iPBM has been demonstrated to have high efficacy for the treatment of vascular, cardiac, and various other pathologic processes, such as tissue repair [22,23].

However, most of the existing articles on the efficacy of iPBM in insomnia, muscle pain, and fatigue are case reports with small sample sizes [14,24]. Based on our previous case reports, we hypothesized that iPBM could improve insomnia for several reasons. First, pain relief was an important factor in improving sleep quality [25]. This was because pain could activate the body’s stress response, which could lead to increased levels of arousal and wakefulness [26]. In addition, physical discomfort made it hard to find a comfortable sleeping position or stay still during the night. Therefore, if iPBM was able to effectively reduce pain and discomfort, this may contribute to improved sleep quality by reducing the level of arousal and allowing the body to relax. The postulations suggested that iPBM may stimulate the release of endorphins, which have a positive impact on mood and relaxation [27,28]. In addition, iPBM was considered to increase blood flow and oxygenation to the affected areas, promoting tissue repair and reducing inflammation [29]. Overall, these postulations suggested that iPBM may promote restful sleep and reduce the risk of waking up during the night due to pain or discomfort. Unfortunately, due to the lack of population verification and quantitative analysis, further research is required to assess the efficacy of iPBM in patients with insomnia, muscle pain, and fatigue.

Previous research indicates iPBM may benefit multiple organ systems by modulating immunity, improving blood cells’ oxygen-carrying capacity, and increasing blood flow [30,31]. Moreover, the low-power laser treatment may enhance red blood cell oxygenation and red blood cell deformability, and thus potentially promote tissue repair and provide pain relief [32]. A few cohort studies have demonstrated that iPBM partially alters red blood cell counts; however, those results were obtained in studies with relatively small cohorts [33,34]. Furthermore, it is still unknown whether iPBM actually improves blood parameters. Therefore, further cohort studies of iPBM treatment that include objective biochemical data are needed.

In consideration of the factors above, we propose that iPBM may relieve symptoms of insomnia, muscle pain, and fatigue by influencing metabolism. Thus, we conducted a controlled analysis of the blood parameters of patients before and after iPBM treatment. Furthermore, we also compared the groups of patients who received ≥10 or 1–9 iPBM treatments to understand whether a higher number of treatments has a more beneficial effect. In addition, we evaluated the outcomes of patients before and after iPBM therapy by assessing their drug use to examine the potential therapeutic efficacy and improve our knowledge of iPBM.

## 2. Methods

### 2.1. Participants

This single-center, retrospective study was conducted in Taiwan. The study design was approved by Kaohsiung Veterans General Hospital Committee on Human Research (IRB number: KSVGH20-CT-16); the requirement for informed consent was waived due to the retrospective study design and minimal risk to participants. All data were deidentified, and no names or identifying information were revealed.

We retrospectively reviewed the KSVGH database and retrieved data on all patients who received iPBM (Taiex He-Ne Laser, YJ-iPBM-5, Bio-iPBM Human Energy Corporation, Kaohsiung, Taiwan) between January 2013 and August 2021. The inclusion criterion was treatment with iPBM selected by charge codes (66026C, 66029J, 66039, and 66040). All iPBM was performed by skilled physiatrists and was indicated for promoting wound healing and relieving pain, and also promoting blood circulation and physiological functions, improving insomnia, metabolism, and immunity, as well as anti-inflammation and anti-infection effects. Patients who complained about pain and insomnia accounted for 70 and 10 percent of the patients we recruited, respectively. Patients with missing data for the three months before the first iPBM treatment or three months after the last iPBM treatment were excluded. 

### 2.2. Intervention of iPBM

**Figure 1:**
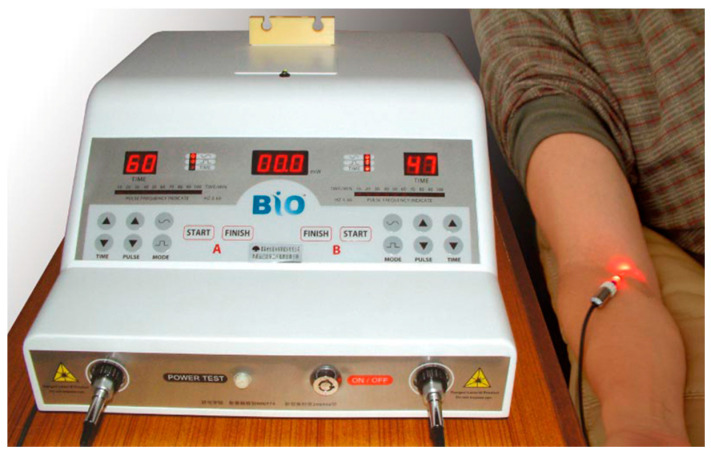
iPBM device and application method of treatment machine.

iPBM is a therapeutic procedure that involves the use of low-level laser light to stimulate various cellular processes and improve blood flow throughout the body. During the procedure, a catheter was inserted into a vein, and a fiber-optic cable was used to deliver visible red light directly into the bloodstream (Figure 1). The red light used in the iPBM treatment had a wavelength of 632.8 nm, an energy output of 2.5 mW, an energy intensity of 1.28 W/cm, a total energy of 9.00 J, an exposure time of 60 min, and an energy density of 4591.84 J/cm^2^. This light could potentially affect cellular metabolism, energy production, and blood flow, which could lead to various therapeutic effects.

The iPBM treatment was administered as a single treatment course over a period of two consecutive weeks on weekdays. There were courses with a rest period of one to three weeks between each course, which took place over two to three months. The treatment was initiated through an optical fiber 0.5 mm in diameter via a phlebotomy cannula to an easily accessible peripheral vein. This approach ensured that almost 100% of the total blood volume was irradiated during each 60 min session. The mean transit time from the regional arm to the brain was less than 30 s, indicating that all the blood was likely irradiated during each session [35]. Although the iPBM treatment required the insertion of a catheter into a vein, it was generally considered a minimally invasive procedure because it did not require any incisions or major surgical interventions. However, catheter insertion could be associated with some discomfort and possible complications, such as bleeding, infection, or vein inflammation.

In summary, iPBM was a minimally invasive procedure that involved the use of low-level laser light to stimulate various cellular processes and improve blood flow throughout the body. The treatment was administered through a catheter inserted into a vein, and almost 100% of the total blood volume was irradiated during each session. While catheter insertion could be associated with some discomfort and possible complications, the therapeutic potential of iPBM may have outweighed these risks for some patients.

### 2.3. Data Collection

All data were retrospectively collected from medical records, for instance, demographics, laboratory data, length of stay, and drug use. We compared the patients’ characteristics, blood test data, and drug use within the three months before their first iPBM treatment and the three months after their last iPBM treatment. In addition, we also compared the blood test data and drug use before and after iPBM treatment in the groups of patients who received ≥10 or 1–9 iPBM treatments. 

The variables assessed included diabetes mellitus (DM), hypertension (HTN), hyperlipidemia, and hematology tests and biochemistry data such as white blood cell count (WBC), hemoglobin (HGB), hematocrit (HCT), platelets (PLT), blood urea nitrogen (BUN), creatinine (Cr), and serum glutamate pyruvate transaminase (GPT). 

### 2.4. Pharmacotherapy Assessment

The assessment of drug use included hypnotic drugs, muscle relaxants, and analgesics. These drugs were selected based on the drugs used by Kaohsiung Veterans General Hospital and the ATC codes (N05C, M03BX, N05BX, N02B, M01AE, and N02AX02). Days of drug use within the 90 days before the first iPBM treatment and 90 days after the last iPBM treatment were determined to assess the efficacy of iPBM therapy. The sums of the days of use of the same types of drugs were divided by 90 as a daily use assessment. 

### 2.5. Statistical Analysis

Demographic and clinical characteristics were summarized for the entire analytical population, divided into the with iPBM therapy group and without iPBM therapy group. They are appropriately expressed as means ± standard deviation or number (percentage). For continuous variables, the comparisons between iPBM therapy group or not were performed using independent samples *t*-test or one-way ANOVA, while for the comparisons between before and after iPBM therapy, paired samples *t*-test or the Wilcoxon rank-sum test was employed. For categorical variables, the comparisons between groups were made using the Chi-squared test or Fisher’s exact test if cells have expected frequencies < 5. All statistical analyses were performed using Statistical Analysis Software (SAS; version 9.4; SAS System for Windows) and SPSS (version 20; SPSS Inc., Chicago, IL, USA). A *p* value of <0.05 was considered statistically significant.

## 3. Results

### 3.1. Patient Characteristics

**Figure 2:**
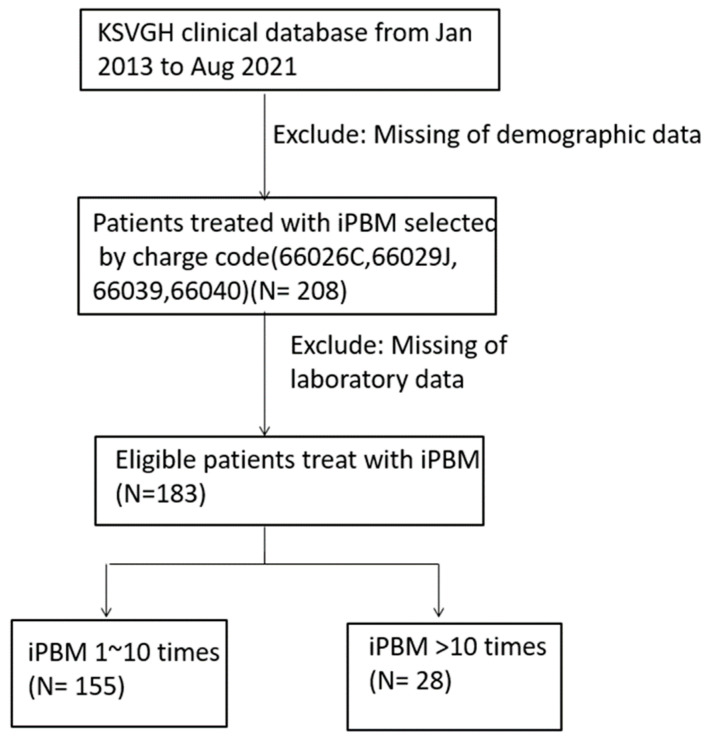
Flowchart.

The subjects and study cohorts were selected according to the process presented in Figure 2. A total of 208 patients who received iPBM therapy between January 2013 and August 2021 were evaluated. We excluded 19 patients whose clinical profiles were not available or had missing data. A total of 183 patients aged 25 to 75 years old (86 males, 97 females; mean age, 60.8 ± 14.9) met the selection criteria and were enrolled in this study. 

We also classified these 183 patients into two groups according to their number of iPBM treatments: 28 patients had ≥10 iPBM treatments, and the other 155 patients had between 1 and 9 iPBM treatments.

Table 1 presents the baseline clinicopathological characteristics and blood data for the 183 patients, and Table 2 summarizes the blood parameters.

### 3.2. Impact of iPBM on Laboratory Data

As shown in Table 2, both HGB and HCT significantly increased after treatment in both the ≥10 and 1–9 iPBM treatment groups (HGB *p* < 0.001 and *p* = 0.046; HCT *p* < 0.001 and *p* = 0.029, respectively). WBC and GPT only decreased significantly after treatment in the 1–9 iPBM treatment group (*p* = 0.010 and *p* < 0.001, respectively), whereas Cr decreased significantly after treatment in both groups (*p* < 0.001).

**Figure 3:**
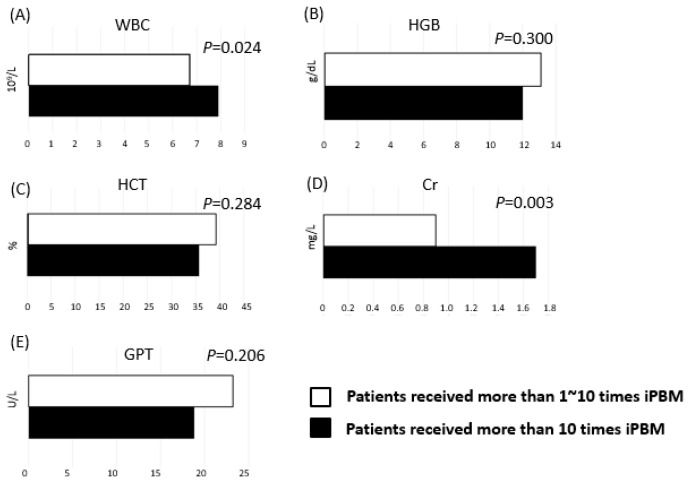
Comparison of the lab data between the <10 and >10 times iPBM groups after the treatment. (**A**) WBC, (**B**) HGB, (**C**) HCT, (**D**) Cr, and (**E**) GPT.

In the comparison of the post-treatment values of the ≥10 and 1–9 iPBM treatment groups, WBC (*p* = 0.024) and Cr (*p* = 0.003) were significantly higher in the patients who received ≥10 treatments; no other parameters were significantly different after treatment between the patients who received ≥10 or 1–9 iPBM treatments (Figure 3).

### 3.3. Pharmacotherapy Analysis

**Figure 4:**
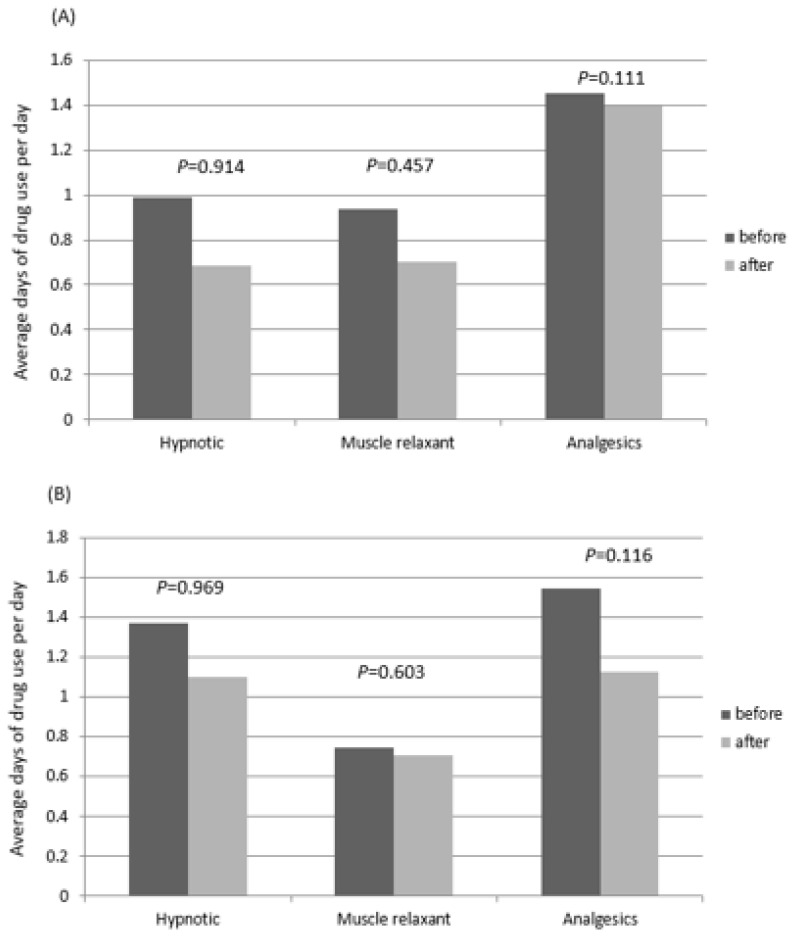
Pharmacotherapy analysis. (**A**) Patients who underwent iPBM treatment 1 to 10 times. (**B**) Patients who underwent iPBM treatment more than 10 times.

As shown in Figure 4, although the days of usage of hypnotic agents, muscle relaxants, and analgesics decreased from 0.987, 0.937, and 1.454 to 0.684, 0.702, and 1.3982, respectively, after treatment in the 1–9 iPBM group, these changes did not reach statistical significance. Similar trends were observed in the ≥10 treatment group.

## 4. Discussion

Over the past 40 years, a number of clinical studies have suggested that iPBM can improve insomnia, metabolism, muscle soreness, and muscle pain; however, the effects of iPBM have not been investigated in cohort studies. To our knowledge, this is the first study to assess the associations between drug use for insomnia or muscle pain and muscle relaxants and the efficacy of iPBM. This study aimed to evaluate the clinical impact of iPBM therapy on the laboratory parameters and drug use of patients with insomnia, muscle pain, and fatigue. We retrospectively analyzed 183 patients who were classified according to the number of treatments. The main finding was that both ≥10 and 1–9 iPBM treatments improved HGB and HCT. However, HGB and HCT were not significantly different after treatment between the groups who received ≥10 or 1–9 iPBM treatments. Furthermore, our medication analysis revealed that iPBM tended to reduce drug use, but these changes were not statistically significant.

We found that HGB and HCT significantly improved after iPBM treatment regardless of the number of iPBM treatments. These findings are similar to, but not exactly the same, as the results of a Russian comparative study of 120 patients with peritonitis [36]. Zimon et al. applied intensive iPBM therapy in 60 patients with peritonitis and found that iPBM stimulated the bone marrow and the organs in which blood elements are stored, and as a result, intensified ejection into the circulation. In fact, these changes in RBCs were associated with better morphometry and electrophoretic mobility. iPBM has been shown to restore normal red blood cell morphology, reduce transition morphology, and increase electrophoretic mobility [37]. Experimental studies in animal models also observed the same results [38,39]. Deryugina et al. conducted a comparative study and found that low-level laser therapy restored RBC phase portraits, improved electrophoretic mobility and osmotic resistance, and increased the RBC count in the peripheral blood of rats [38]. Moreover, laser light was used as a marker for RBC viability, and thus increased the viability of RBCs [40]. While the exact mechanisms underlying the potential effects of iPBM on hematocrit (HCT) and hemoglobin (HB) levels are not well understood, there are several hypotheses. One hypothesis is that the low-level laser light used in these treatments may stimulate the production of erythropoietin, a hormone that plays a key role in the production of red blood cells. This, in turn, could lead to an increase in HCT and HB levels. Another hypothesis was that some researchers have suggested that the laser light may stimulate the production of nitric oxide, a signaling molecule that plays a key role in regulating blood flow and blood vessel dilation, which could also contribute to improved blood flow and oxygen delivery, leading to an increase in HCT and HB levels [41].

However, HGB and HCT were not significantly different after treatment between the groups who received ≥10 or 1–9 iPBM treatments. Patients who received fewer than 10 iPBM treatments had higher HGB and HCT before treatment; thus, the lack of difference between groups who received ≥10 or 1–9 iPBM treatments may be due to the fact that the >10 iPBM group had a more severe disease status. In fact, average HGB and HCT were lower before treatment in the >10 group (11.1 and 32.9, respectively), compared to the 1–9 iPBM group (12.3 and 36.6, respectively). More than 10 courses of iPBM increased the average HGB and HCT by 1 and 2.8, which was slightly greater than the amount of change in the <10 iPBM treatment group. In addition, some patients who received a small number of courses of iPBM received other treatments, such as blood transfusion and/or drug therapies, including berberine, eperisone hydrochloride, and steroids, which can also improve HGB and HCT [42,43,44]. Therefore, although it was not possible to perform stratified analysis because of the wide variety of drugs, our results demonstrate that >10 iPBM treatments might lead to better effects than <10 treatments, and that iPBM may even represent an alternative treatment to drugs.

In the study, we also observed a decrease in the average creatine levels among the 183 subjects after receiving intravascular photobiomodulation (iPBM) treatment. The creatine levels decreased from 1.1 to 0.9 and 2.0 to 1.7 in both the ≥10 and 1–9 iPBM treatment groups, respectively (*p* < 0.001). The finding suggested that iPBM treatment may be an effective intervention for reducing creatine levels. The underlying mechanism was still unclear. However, previous studies have suggested that iPBM treatment can stimulate the production of nitric oxide (NO), a potent vasodilator that can increase blood flow and improve tissue oxygenation [41,45]. This increased blood flow and oxygenation may help to enhance cellular metabolism and reduce inflammation, which could potentially lead to lower creatine levels. Additionally, iPBM treatment may also have a direct effect on the mitochondria within cells, leading to improved energy production and metabolism, which could also contribute to reduced creatinine levels [46].

We further investigated whether iPBM therapy can reduce the use of various commonly prescribed drugs. There was no significant reduction in the days of drug use after iPBM, despite the patients’ reports of symptomatic relief. This may be due to several reasons. First, due to the accessibility of Taiwan’s medical and health insurance system, patients and doctors largely prescribe medicines according to their own habits, even if the patient’s symptoms reduce. Moreover, theoretically, we originally assumed that patients would have higher medicine use several tens of days after the last iPBM treatment due to ‘therapy withdrawal’ or the ‘rebounding effect’. There was a trend toward lower drug use after iPBM treatment, regardless of the number of treatments, compared to pre-treatment. A similar result was also noted in the recent study by Wu et al. [47], in which drugs and iPBM both led to significant improvements among patients with fibromyalgia. Other studies have also shown that iPBM-treated patients function similarly to patients with certain diseases treated with other drugs [48,49,50]. For example, we reported a case of a middle-aged woman who survived vaccine-induced GBS but had persistent paresthesia and pain affecting her sleep quality. The patient was taking Gabapentin and Xanax before our intervention, and the Pittsburgh Sleep Quality Index (PSQI) on admission revealed a score of 12 out of 21. However, The PSQI was re-tested on the day of discharge after five courses of ILIB and showed marked improvement, with a score of 7 [49]. Hence, we consider that iPBM has a positive effect on reducing drug use. However, the improvements in the patient’s symptoms were not fully represented by the number of days of drug use, and only a small number of patients received more than 10 iPBM treatments. Larger studies using scales for specific symptoms are needed to explore the changes in symptoms after iPBM treatment more precisely.

This study has some inherent limitations. Firstly, as this was a retrospective analysis, the data for several important variables were lost or insufficient, such as the homeostasis model assessment–insulin resistance index and some of the pre- and post-treatment variables. Second, we only included data on medicines from the KSVGH database; some patients may have obtained medicines from other providers not included in the database. However, we believe that the risk of bias was small, because most of the patients were treated and followed-up by the same physicians. Thirdly, we did not perform a control group analysis in the study. However, it is important to note that the study was designed as an exploratory investigation of the intervention’s potential efficacy, and the lack of a control group was a conscious decision made to maximize the sample size and feasibility of the study. In the future, we plan to conduct a controlled trial to further investigate the intervention’s effects and better understand the underlying mechanisms. Finally, only patients from Taiwan were enrolled in this study. It is necessary to provide additional studies to confirm whether these trends also occur in other cohorts from Asia and Western countries.

In conclusion, the present study confirmed that iPBM therapy is an efficient, beneficial, and feasible strategy to increase HGB and HCT in patients with insomnia, muscle pain, and fatigue. Moreover, this was the first study to assess the association between iPBM therapy and drug use for insomnia or muscle pain and muscle relaxants. Although our results do not support the suggestion that iPBM reduces the days of drug use, a trend towards decreased drug use was observed. These findings provide important insights that will be useful for the development of rehabilitation interventions. Moreover, it is suggested that iPBM may be a novel alternative treatment in the future.

## 5. Conclusions

iPBM therapy is an efficient, beneficial, and feasible treatment that increases HGB and HCT. While the results of this study do not support the suggestion that iPBM reduces drug use, further larger studies using symptom scales are needed to confirm the changes in insomnia and muscle soreness after iPBM treatment.

## Figures and Tables

**Table 1:** Characteristics of the 183 patients with iPBM therapy.

Variable	Patients with iPBM (N = 183)
Male	86 (47%)
Age	60.8 ± 14.9
BMI	26.4 ± 16.2
DM	59 (32%)
HTN	21 (12%)
Hyperlipidemia	33 (18%)
Depression	6 (3.3%)
Insomnia	18 (10%)
PSTD	0 (0%)
WBC	11.3 ± 5.9
HGB	12.1 ± 2.1
HCT	36.1 ± 5.9
PLT	310.0 ± 120.7
Cr	1.2 ± 1.4
GPT	40.3 ± 36.3

**Table 2:** Comparison of the lab data between the before and after iPBM.

Variable	iPBM Times	Before	After	*p*-Value
WBC	<10	11.1 ± 5.9	6.7 ± 2.2	0.010
	>10	12.5 ± 5.7	7.9 ± 3.9	0.221
HGB	<10	12.3 ± 2.0	13.1 ± 1.5	<0.001
	>10	11.1 ± 1.9	12.0 ± 1.8	0.046
HCT	<10	36.6 ± 5.8	39.2 ± 4.4	<0.001
	>10	32.9 ± 5.8	35.7 ± 5.0	0.029
Cr	<10	1.1 ± 0.8	0.9 ± 0.4	<0.001
	>10	2.0 ± 3.2	1.7 ± 2.8	<0.001
GPT	<10	40.6 ± 36.4	23.2 ± 14.3	<0.001
	>10	48.5 ± 54.0	18.8 ± 7.8	0.878

## Data Availability

All data were deidentified, and no names or identifying information were revealed.

## Abbreviations

Intravascular photobiomodulation (iPBM), diabetes mellitus (DM), hypertension (HTN), red blood cell (RBC), white blood cell (WBC), hemoglobin (HGB), hematocrit (HCT), platelets (PLT), blood urea nitrogen (BUN), creatinine (Cr), glutamate pyruvate transaminase (GPT), and adenosine triphosphate (ATP).
